# Characteristics, Risk Factors, and Outcomes in Acute Kidney Injury Patients: A Retrospective Cross-Sectional Study, Palestine

**DOI:** 10.1155/2024/8897932

**Published:** 2024-04-08

**Authors:** Abdallah Damin Abukhalil, Haya Alyazouri, Reem Alsheikh, Hadeel Kahla, Minna Mousa, Hosniyeh Ladadweh, Ni'meh Al-Shami, Yousef Sahoury, Hani Naseef, Abdullah Rabba

**Affiliations:** Department of Pharmacy, Faculty of Pharmacy, Nursing and Health Professions, Birzeit University, West Bank, State of Palestine

## Abstract

**Background:**

Acute kidney injury (AKI) is a major medical problem affecting patients' quality of life and healthcare costs.

**Objectives:**

This study evaluated the severity, risk factors, and outcomes of patients diagnosed with acute kidney injury (AKI), including community-acquired AKI (CA-AKI) and hospital-acquired AKI (HA-AKI), who were admitted to tertiary institutions in Palestine.

**Methods:**

This retrospective cross-sectional study was conducted at multiple tertiary care hospitals in Palestine by reviewing patient charts from January 2020 to March 2023. The study included all patients aged ≥18 years who were admitted to the hospital and diagnosed with AKI at admission (CA-AKI) or who developed AKI 48 hours after admission (HA-AKI). Patients with incomplete medical records and those with no reported creatinine levels during their stay, pregnant women, kidney transplant patients, and end-stage renal disease patients were excluded. Data were analyzed using SPSS v22.0. The incidence of AKI in each group was compared using the chi-squared test.

**Results:**

This study included 259 participants. HA-AKI was present in 27.3% of the patients, while CA-AKI was 72.7%. The most common stage among patients was stage 3 (55.7%, HA-AKI) (42.9%, CA-AKI), and the most common comorbidity contributing to AKI was CKD. NSAIDs, ACE-I/ARBs, and DIURETICs were the most nephrotoxic drugs contributing to AKI. Patients with hyperphosphatemia, hyperkalemia, severe metabolic acidosis, or stage 3 AKI require renal replacement therapy. In addition, our findings revealed a significant association among AKI mortality, age, and heart disease.

**Conclusion:**

CA-AKI was more prevalent than HA-AKI in Palestinian patients admitted for AKI. Risk factors for AKI included diabetes, CKD, and medications (antibiotics, NSAID, diuretics, and ACE-I/ARB). Preventive measures, medication management, and disease state management are necessary to minimize AKI during hospital admission or in the community.

## 1. Introduction

AKI is a life-threatening syndrome that may lead to serious long-term complications such as chronic kidney disease or progression to end-stage renal disease. It affects more than 13 million people annually, resulting in 1.7 million deaths, affecting 1 in 5 hospitalized patients, and reaching up to 50% of critically ill patients [1, 2]. Furthermore, more than 50% of patients transferred to the intensive care unit (ICU) develop acute renal impairment during their first transfer hours [1, 3–5]. AKI is associated with increased healthcare costs and length of hospital stay [6]. Therefore, strategies for detecting, preventing, and facilitating kidney recovery are required.

Acute kidney injury (AKI) is a sudden loss of kidney function that results in the retention of urea and other nitrogenous waste products, causing an imbalance of plasma fluid and electrolytes [3, 7]. Kidney Disease Improving Global Outcomes (KDIGOs) classify kidney injury into three stages based on serum creatinine levels and urine output [4]. Furthermore, AKI can be classified based on its etiology as prerenal, intrinsic, or postrenal [5].

Risk factors for AKI have been well documented in the literature, including age, diabetes, cardiovascular disease, chronic kidney disease, and nephrotoxic medications [8, 9]. Diabetes being the most common risk factor account for 50% of patients with AKI. Patients with heart failure have a 26% risk of developing AKI compared with 14% in those without heart failure [10]. The incidence of AKI in patients diagnosed with sepsis ranges from 11% to 70% [11]. Moreover, obstructive nephropathy may have a 5–10% impact on AKI [12]. Many commonly prescribed medications have been linked up to 50% increase in the risk of AKI, especially nephrotoxic drugs when used in combinations such as renin-angiotensin system inhibitors, diuretics, nonsteroidal anti-inflammatory drugs (NSAIDs), aminoglycosides, vancomycin, and radiocontrast agents [13–15].

Differences in the etiology, risk factors, and prevalence of community-acquired AKI (CA-AKI) and hospital-acquired AKI (HA-AKI) have been reported in different regions worldwide [16, 17]. Community-acquired AKI (CA-AKI) has been reported as the dominant form of AKI [18]. Both types are associated with different risk factors, presentation, and epidemiological profiles. Furthermore, controversial reports on prognoses, mortality rates, renal complications, length of hospital stay, and healthcare costs have been published [1, 19]. In a study conducted in the United Kingdom, CA-AKI was more severe than HA-AKI, although both had similar risk factors. CA-AKI patients had lower mortality rates despite having risk factors similar to those of patients with HA-AKI; furthermore, patients with CA-AKI had better survival rates and shorter hospital stays [20]. Furthermore, in a meta-analysis, CA-AKI patients had a good prognosis and improved clinical manifestations with fewer reported ICU admissions, hospital stays, and mortality rates [21]. It has been reported in the literature that AKI features and outcomes differ by geographic and socioeconomic regions [22].

Regional studies on AKI prevalence, risk factors, and settings are limited. A recent study in Turkey revealed that nephrotoxic drugs are the most common etiology of AKI with a 24% death rate after 6 months of diagnosis, and a study in Saudi Arabia on HA-AKI reported serious complications and minimal renal recovery [23, 24]. To the best of our knowledge, no studies have been conducted in Palestine to explore this topic; therefore, this study aimed to evaluate the severity, risk factors, outcomes, and prevalence of CA-AKI and HA-AKI in patients diagnosed with AKI at tertiary care hospitals in Palestine. The findings of this study will be used to establish preventive measures to ensure patient safety, optimize medication therapy, and prevent complications.

## 2. Material and Methods

### 2.1. Study Design and Population

This retrospective cross-sectional study was conducted at multiple tertiary care hospitals in Palestine by reviewing the patient data from January 2020 to March 2023. The study included all patients aged ≥18 years who were admitted to the hospital and diagnosed with AKI at admission (CA-AKI) or who developed AKI 48 hours after admission (HA-AKI). Patients with incomplete medical records, those lacking reported creatinine levels during their stay, pregnant women, kidney transplant patients, or end-stage renal disease patients were excluded.

### 2.2. Data Collection

The data collection form was based on a review of other AKI studies. Data were gathered from patient records and entered into Google form sheets. This form was divided into five sections. In the first section, patient demographics, age, gender, social history, and admission, and discharge dates were recorded. The second section included past medical history (acute and chronic medical conditions), and the third section included AKI-related information, medications before and during admission, nephrotoxic drug exposure, and date of AKI diagnosis. The fourth section included laboratory data (serum creatinine level and BUN ratio), stage categories (stages 1, 2, and 3), and AKI classification (prerenal, intrinsic, and postrenal). The last section included AKI complications, metabolic acidosis, electrolyte imbalance, critical care admission, and mortality.

### 2.3. AKI Classification

According to the Kidney Disease Improving Global Outcomes (KDIGO) approach, the patient population was divided into three stages depending on the serum creatinine level and urine output. Stage 1 is defined as an increase in SCr of less than 0.3 mg/dL within 48 hours or 1.5 to 1.9 times baseline SCr within 7 days. Stage 2 shows an increase in SCr of 2.0 to 2.9 over the baseline SCr. Stage 3 is defined as an increase in SCr of more than 3.0 times the baseline, an increase in SCr of more than 4.0 mg/dL, or the start of renal replacement therapy despite having previously reached a KDIGO stage.

### 2.4. Statistical Analysis

Data were analyzed using SPSS v22.0. The data were imported, cleaned, and recorded as needed. Continuous variables are presented as mean ± SD or median (25th–75th percentile), as appropriate. Categorical variables were presented as proportions. Pearson's chi-square test and Fischer's exact test were performed to compare the CA-AKI and HA-AKI groups and to assess the differences in complications. For significant results, binary logistic regression (the entered model) was performed to identify confounders. Odds ratios (ORs) with 95% confidence intervals (CIs) were also calculated. The hypotheses were two-tailed, and a *P* value less than 0.05 was considered statistically significant.

### 2.5. Ethical Considerations

The study design was approved by the Ethical Committee of Birzeit University (BZUPNH 2203). Informed consent was obtained from the official authority to conduct this study. The information obtained was utilized purely for research and kept confidential. This study adhered to all ethical requirements.

## 3. Results

### 3.1. Demographics, Classification, and Characteristics

In total, 259 patients met the inclusion criteria. Approximately half of the participants (54.4%) were male. The most prevalent AKI stage was stage 3 (120 patients, 46.3%), followed by stage 2 (76 patients, 29.3%), and the last stage was stage 1 (63, 24.3%).

**Figure 1:**
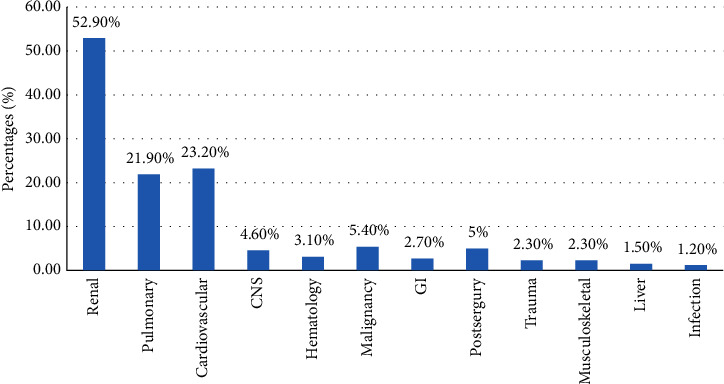
Diagnosis on admission (CNS: central nervous system; GI: gastrointestinal) (*N* = 259).

The diagnoses on admission are presented in Figure 1. The most common diagnosis for patients with AKI on admission was renal disease (52.9%), followed by cardiovascular (23.2%) and pulmonary (21.9%) diseases; infections were the least common diagnoses (1.2%).

A total of 189 participants (72.7%) were admitted to the hospital with AKI (CA-AKI), whereas 70 (27.3%) developed AKI during hospitalization (HA-AKI). Demographic differences between the CA-AKI and HA-AKI groups are shown in Table 1. There was no significant difference in the possibility of developing CA-AKI or HA-AKI and patients' age, gender, or AKI stage. Prerenal etiology was significantly more common among CA-AKI patients (54.5%) than HA-AKI patients (41.4%) (*P*=0.006). A comparison of various comorbidities in patients with HA-AKI and CA-AKI revealed a significantly higher prevalence of CKD in patients with CA-AKI (32.3%) than in those with HA-AKI (11.4%; *P*=0.001). No difference was found in the proportions of patients with diabetes mellitus, anemia, liver disease, or heart disease between the two groups.

### 3.2. Drug-Induced AKI

AKI risk medications included NSAIDs, antibiotics, ACE-I/ARBs, diuretics, and SGELT2 (dapagliflozin). As shown in Table 1, prior exposure to nephrotoxic drugs was significantly higher among CA-AKI patients (77.8%) than among HA-AKI patients (44.3%) (*P*=0.001). Prior exposure to NSAIDs, ACE-I/ARBs, and diuretics was significantly higher among CA-AKI patients than HA-AKI patients (NSAIDs 49.7% vs. 18.6%, *P*=0.001, ACE-I/ARBs 32.8% vs.18.6%, *P*=0.025, and diuretics 39.2% vs. 15.7%, *P*=0.001). There was no difference in the frequencies between the two groups, and the number of medication patients were exposed to before admission. During admission, NSAID administration was significantly more prevalent in patients with CA-AKI (48.7%) than in those with HA-AKI (31.4%; *P*=0.01). In contrast, antibiotic use was higher in patients with HA-AKI (90%) than in those with CA-AKI (49.7%; *P* < 0.001).

### 3.3. AKI Outcomes

#### 3.3.1. Recovery

The proportions of patients recovering from baseline serum creatinine levels, persistent AKI, and renal replacement therapy did not differ between the CA-AKI and HA-AKI groups. ICU transfer frequency was significantly higher in the HA-AKI group (37.1%) than in the CA-AKI group (18.5%, *P*=0.002).

#### 3.3.2. Complications

**Figure 2:**
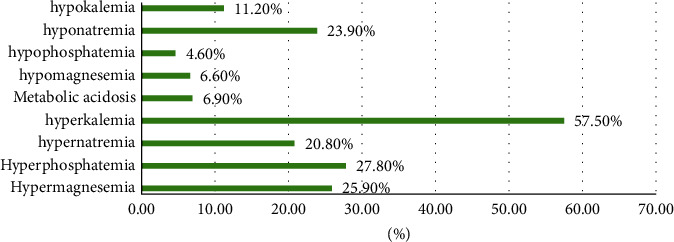
AKI complications.

The electrolyte imbalance associated with AKI is shown in Figure 2. Hyperkalemia (57.5%) was the most common electrolyte disturbance, followed by hyperphosphatemia (27.8%), hypermagnesaemia (25.9%), and hyponatremia (23.9%).

Binary logistic regression results, presented in Table 2, revealed that among patients with AKI, increasing age and heart disease were two factors associated with an increased risk of mortality (*P* ≤ 0.001 and *P*=0.003, respectively). A one-year increase in age was associated with an approximately 6% increase in mortality risk. Additionally, the risk of mortality increases by 70% in patients with heart disease. No difference in the risk of mortality was assessed between patients with other comorbidities, those who developed different complications, or those with different AKI stages.

As shown in Table 3, binary logistic regression analysis revealed a significant association between renal replacement therapy and AKI complications, including hyperphosphatemia and metabolic acidosis (*P*=0.045 and *P* < 0.016, respectively). Patients who develop hyperkalemia or metabolic acidosis are at a higher risk of undergoing renal replacement therapy, by 68% and 82%, respectively. Furthermore, significant associations were found between renal replacement therapy and stage 3 diagnosis (*P*=0.019); patients diagnosed with stage 3 disease were at a 38-fold higher risk of receiving renal replacement therapy. The results revealed that a one-unit increase in baseline SrCr levels was associated with a two-fold increase in the risk of transfer to HD.

## 4. Discussion

In this study, we explored essential data and new information regarding AKI diagnosis, prevalence, patient characteristics, AKI settings (HA-AKI and CA-AKI), and complications. Several factors, including age and diseases such as diabetes, CKD, low blood pressure, and heart disease, as well as taking some medications, contribute to AKI [25].

### 4.1. Patient Characteristics

In this study, CA-AKI was predominant compared with HA-AKI, although both shared similar risk factors and patient characteristics, including age, sex, stage, and comorbidities, with no significant differences. A higher prevalence of CA-AKI has been reported in many developing countries, and a similar finding was reported in a large Taiwanese adult retrospective cohort study [18, 26, 27]. The median age of patients diagnosed with AKI was 65 years. Furthermore, studies have shown that the risk of developing acute renal failure is three times higher in patients aged >65 years than in younger patients [28]. Increased age has been reported as a risk factor in AKI patients because of the physiological and structural changes in the kidneys with aging, which reduce nephron mass and glomerular filtration rate with an increased tendency towards cellular apoptosis [29]. The results showed a strong association between age and mortality (*P* < 0.001). It is widely recognized that the elderly have a higher incidence of AKI, poor prognosis, and a higher mortality risk than other age groups. Elderly patients have frequent comorbidities, including diabetes, hypertension, and heart failure, and take many drugs; this can be associated with the severity and complexity of their condition and mortality [30, 31].

The physiological changes related to age require caution in medication use and dosage and more frequent monitoring of efficacy and toxicity. Furthermore, tools have been established to ensure appropriate prescribing for the elderly that should be incorporated into clinical practice, such as the BEERS list, which includes medication doses that need to be reduced or medications that should be avoided in the elderly [32].

Among the 259 patients who developed AKI, 54.4% were male, and there was no relationship between gender and either CA-AKI or HA-AKI. Some studies have reported the presence and development of AKI in males due to the presence of androgens, which are essential factors that contribute to the destruction and disruption of kidney function by disrupting hemodynamics, which can be due to the more resistant efferent arterioles. In females, during the menstrual cycle, estrogen acts as a protective and therapeutic agent for the kidneys; however, this short-term effect decreases with age [29]. Furthermore, in a recent Systematic Review and Meta-Analysis, male gender was one of the many risk factors that placed COVID-19 patients at greater risk of developing AKI (OR: 1.74 (1.47, 2.05), *P* < 0.05) [33].

### 4.2. Comorbidities

In this study, there was no association between comorbidities such as diabetes, heart disease, liver disease, and anemia and the incidence of CA-AKI or HA-AKI, and patients with these comorbidities were more likely to develop AKI [34]. A very similar finding was illustrated in a Moroccan study where various comorbid conditions had equal prevalence among patients with CA-AKI and HA-AKI [35]. Furthermore, patients with CKD are at an increased risk of developing AKI, which is significant in CA-AKI. In CKD patients, the kidney loses its response or tolerates stress such as infection, dehydration, nephrotoxic drugs, and comorbidities, which decreases the threshold for developing AKI affecting renal function or perfusion [36, 37]. In this study, CKD was a risk factor for CA-AKI, and in this study, it was clear that patients in the community setting have significant exposure to nephrotoxic medications, which calls for an increase in awareness of factors that might cause exacerbation of renal function.

## 5. AKI Stage

AKI stage is a determining factor of prognosis, with the higher stage having a poorer outcome, stage 1 treated in primary care, and stage 3 treated in secondary care [38]. In this study, stage 3 was the predominant stage regardless of the AKI setting, with approximately 50% of the participants having stage 3 AKI. A similar finding was reported in a Sudanese study, and a smaller rate was reported in Chinese studies [39, 40]. This alarming finding may explain the poor prognosis, poor outcomes, and extended length of hospital stay. The late-stage presentation may be due to lack of access to care, lack of follow-up, understanding of AKI, and asymptomatic patients. Therefore, educating at-risk patients on the signs, symptoms, and aggravating factors of AKI is of utmost importance. Furthermore, detailed patient assessments and physical examinations when suspecting acute renal insufficiency will promptly identify and treat the causes [41].

### 5.1. AKI Etiology

Prerenal was the most prevalent etiology in the sample; almost half of the patients had prerenal AKI, which was significantly higher in the CA-AKI group. A similar finding in a prospective study in a tertiary care center in China found that prerenal was the most frequent cause of AKI, occurring in 27.7% of the cases [42]. Researchers in a recent regional study in Turkey that included 776 patients diagnosed with AKI found that prerenal AKI was the most prevalent etiology with high exposure to nephrotoxic medication, similar to our finding [24]. Prerenal AKI occurs due to kidney hypoperfusion, which can be related to a decline in cardiac output volume and renal perfusion due to different conditions such as bleeding, severe vomiting or diarrhea, dehydration, or diuretic use. This finding can also be explained by the significantly higher exposure to nephrotoxic drugs such as NSAIDs, ACE-I/ARBs, and diuretics in the CA-AKI group. A very similar finding was also reported in a Taiwanese study [18].

In prerenal AKI, many changes occur in the afferent and efferent arterioles due to the use of different drugs, such as ACE inhibitors or ARBs, which decrease efferent arteriolar resistance. Nonsteroidal anti-inflammatory drugs (NSAIDs) affect the renal production of prostaglandin and cause vasodilation of the afferent arterioles, which generally need to be constricted [43].

### 5.2. Nephrotoxic Drugs

The drug classes associated with AKI in this study were similar to those reported previously. This finding is very similar to a study conducted in China, where the prevalence of nephrotoxic drugs was 57% and much higher in Taiwan [44, 45]. Evidence suggests that exposure to at least one nephrotoxic drug is associated with AKI and its severity [46].

ACE-I is one of the most effective drugs for the management of many comorbid diseases such as hypertension and congestive heart failure. However, appropriate dosage and monitoring at initiation and during treatment are essential to prevent complications. ACE-I/ARBS alters the glomerular filtration rate, affects interglomerular hemodynamics, and causes AKI [47]. NSAIDs are widely used as over-the-counter (OTC) medications. Surprisingly, NSAIDs, which are contraindicated in patients with AKI, are frequently used in patients with AKI during admission. In a regional cohort study in Saudi Arabia, NSAIDs were the most common cause of in-hospital AKI [48].

NSAIDs can cause a poor prognosis, mainly when used with other medications such as ACE and diuretics during AKI [49]. A recent study in Palestine among college students reported very high use of NSAID and suggested increasing awareness of adverse effects associated with NSAID [50].

ACE-I, ARB, and NSAIDs have a nephrotoxic effect on vasodilation of the efferent arteriolar and vasoconstriction of the afferent renal arteriolar, respectively [47]. A national cohort study showed that ICU survivors exposed to at least one nephrotoxic agent needed renal replacement therapy [30].

Antibiotics were significantly associated with HA-AKI during hospitalization. This finding is in line with other reports in the literature, where antibiotics induce renal toxicity through different mechanisms that alter intraglomerular hemodynamics or cause inflammatory changes in the renal tubules [8, 51]. Cephalosporin, aminoglycoside, bacitracin, and vancomycin are associated with an increase in the risk of acute kidney injury (AKI) [52]. Medication management and appropriate renal dosing and monitoring, especially in patients with multiple comorbidities and multiple medications that affect renal function, are essential to prevent AKI and reduce hospital stay.

### 5.3. AKI Outcomes

The outcomes of AKI management include recovery to baseline, the need for RRT, ICU transfer, and mortality. In this study, HA-AKI patients had a significantly higher ICU transfer rate than CA-AKI patients did. A similar finding was reported in a regional multicenter Egyptian cohort study conducted in four teaching hospitals in Alexandria, where 39.6% of patients with AKI were referred to intensive care [53]. On the other hand, a study in Pakistan revealed higher mortality among CA-AKI patients [54].

The mortality rates in this study significantly increased with age and in the patients diagnosed with heart disease. Interrelations between the kidney and the heart are well recognized, and under normal circumstances, any dysfunction in one organ will cause another to compensate and maintain adequate perfusion in the body; however, in renal or heart disease, there is a dysfunction in this feedback mechanism leading to negative outcomes and poor prognosis; therefore, it has been reported that an increase in serum creatine will negatively affect the prognosis of heart failure [55].

A similar retrospective study was conducted at a tertiary teaching hospital in the Khartoum Study. Osman et al. reported lower overall mortality rates with higher mortality rates associated with age, liver disease, and sepsis [39].

The Improving Global Outcomes (KDIGO) guidelines classify AKI according to the severity of kidney damage in three stages. As the stage increases, the severity increases, the risk of developing acid-base and electrolyte imbalances increases, and the need for RRT, which was evident in this study, hyperkalemia, hyperphosphatemia, and metabolic acidosis was significantly associated with the need for RRT [43, 56]. Metabolic acidosis has systemic effects that disrupt cellular and organ function and lead to adverse complications of AKI, as reported by many studies [57–59]. Electrolyte disturbances, such as hyperkalemia, hypermagnesemia, and metabolic acidosis, were significantly associated with higher mortality rates. Patients with hyperkalemia have renal tubular abnormalities and severe stages; furthermore, hyperkalemia increases the risk of arrhythmias, increasing the risk of mortality [60, 61].

Hyperkalemia is highly associated with abnormalities in electrocardiograms (ECG) and arrhythmias. Furthermore, hyperphosphatemia increases the risk of secondary hyperthyroidism and cardiovascular calcination; prompt treatment is essential and lifesaving [62, 63]. Severe metabolic acidosis can affect the cardiovascular system, making it a crucial indicator for starting RRT [64]. The STARRT-AKI study, which involved 3019 patients from different countries, focused on the outcome of early initiation of RRT in stages 2 and 3 of AKI and found that early initiation of therapy did not affect the mortality rate. In this study, 30.8% of stage 3 patients need emergency RRT, and the mortality rate was higher among these patients, as illustrated by the START study that significantly ill patients might not have a reduced mortality rate when undergoing dialysis [65].

## 6. Limitations/Strength

This is the first study performed in multiple hospitals in Palestine and can serve as a core study for future research in this field. AKI was assessed using the Kidney Disease Improving Global Outcome (KIDGO) guidelines. This study provides information on the occurrence of AKI in different settings (hospital vs. community) and is the first to compare the differences between CA-AKI and HA-AKI patients in Palestine. In addition, information on the outcomes (dialysis or mortality) of acute renal impairment was also collected. This study has several limitations, including a small sample size and limited generalization of the results. The study's retrospective design has limitations: retrospective studies only established an association, not a direct cause-effect relationship with risk factors and outcome, and data were collected from medical charts, where patients with missing information or documentation that could affect the results were excluded. Furthermore, neither the healthcare provider nor the patient was contacted, and information was only obtained from medical charts. This calls for prospective and interventional long-term studies to further explore AKI risk factors, etiology prevention, and management in Palestine.

## 7. Conclusion

In this study, we explored the factors associated with AKI. CA-AKI was more prevalent than HA-AKI in Palestinian patients diagnosed with AKI at a tertiary care hospital in Palestine. Nephrotoxic medications (antibiotics, NSAID, diuretics, and ACE-I/ARB) were significant risk factors for AKI. Mortality rates increase with age in patients with congestive heart failure. Stage 3 and prerenal etiology were the most common presentations. The findings of this study highlight the need to take appropriate measures to increase awareness among patients and healthcare providers of risk factors associated with kidney injury in a community or hospital setting. The healthcare system in Palestine faces many challenges due to the lack of resources, such as hospital beds and dialysis units, adding more stress and responsibilities to healthcare providers; therefore, a thorough patient evaluation for early diagnosis and detection of AKI during admission or office visits, addressing modifiable risk factors for AKI, lead to an early intervention to prevent complications, adverse outcomes, hospital stay, and admission. Furthermore, the community pharmacist, also available in the community and serving as a healthcare provider in medication management, is responsible for ensuring the appropriate use of commonly prescribed and utilized nephrotoxic medications to decrease drug-induced AKI.

## Figures and Tables

**Table 1:** Baseline characteristics of patients (*N* = 259).

Variables	Categories	Total *n* (%)	CA-AKI *n* (%)	HA-AKI *n* (%)	*P* value
		259	189 (72.7)	70 (27.3)	
Age (median, 25^th^–75^th^) OR (95% CI)		65 (53–75) 0.98 (0.97–1.005)			0.16

Gender	Male	141 (54.4)	108 (57.1)	33 (47.1)	0.15
	Female	118 (45.6)	81 (42.9)	37 (52.9)	

Stages	Stages 1	63 (24.3)	53 (28)	10 (14.3)	0.05
	Stages 2	76 (29.3)	55 (29.1)	21 (30)	
	Stages 3	120 (46.3)	81 (42.9)	39 (55.7)	

Classification	Prerenal	132 (51)	103 (54.5)	29 (41.4)	**0.006**
	Intrinsic	87 (33.6)	65 (34.4)	22 (31.4)	
	Postrenal	40 (15.4)	21 (11.1)	19 (27.1)	

Comorbidities	Diabetes	134 (51.7)	104 (55)	30 (42.9)	**0.08**
	CKD	69 (26.6)	61 (32.3)	8 (11.4)	**0.001**
	Liver diseases	14 (5.4)	10 (5.3)	4 (5.7)	0.89
	Anemia	16 (6.2)	10 (5.3)	6 (8.6)	0.33
	Heart diseases	81 (31.2)	59 (31.3)	22 (31.5)	0.57

Drugs during admission	NSAIDS	114 (44)	92 (48.7)	22 (31.4)	**0.01**
	Antibiotic	157 (60.6)	94 (49.7)	63 (90)	**<0.001**
	ACE-I/ARBs	55 (21.2)	44 (23.3)	11 (15.7)	0.18
	Diuretic	127 (49)	90 (47.6)	37 (52.9)	0.45
	SGLET2 (Dapagliflozin)	7 (2.7)	5 (2.6)	2 (2.9)	0.92

Prior exposure to nephrotoxic drugs	Yes	178 (68.7)	147 (77.8)	31 (44.3)	**<0.001**
	No	81 (31.3)	42 (22.2)	39 (55.7)	

Prior nephrotoxic drug count	1	67 (25.9)	54 (36.7)	13 (41.9)	0.86
	2	80 (30.9)	67 (45.6)	13 (41.9)	
	3 or more	31 (12)	26 (17.7)	5 (16.1)	

Prior nephrotoxic drug type	NSAIDS	107 (41.3)	94 (49.7)	13 (18.6)	**<0.001**
	Antibiotic	85 (32.8)	24 (12.7)	12 (17.1)	0.35
	ACE-I/ARBs	75 (29)	62 (32.8)	13 (18.6)	**0.025**
	Diuretic	85 (32.8)	74 (39.2)	11 (15.7)	**<0.001**

AKI outcome	Recover serum creatinine baseline	133 (51.4)	102 (54)	31 (44.3)	0.16
	Persistent AKI	76 (29.3)	56 (29.6)	20 (28.6)	0.86
	Renal replacement therapy	46 (17.8)	30 (15.9)	16 (22.9)	0.19
	Transfer to ICU	61 (23.6)	35 (18.5)	26 (37.1)	**0.002**

Mortality related to AKI	Yes	36 (13.9)	26 (13.8)	10 (14.3)	0.91
	No	223 (86.1)	163(86.2)	60 (85.7)	

CA, community-acquired, HA, hospital-acquired; AKI, acute kidney injury; CKD, chronic kidney disease; heart disease, heart failure/ischemic heart diseases/coronary artery diseases; renal replacement therapy: hemodialysis, intensive care units (ICUs); NSAIDS: nonsteroidal anti-inflammatory drugs; SGLT2: sodium-glucose cotransporter-2 inhibitors ACI; ARBS, angiotensin-converting enzyme inhibitor/angiotensin receptor blockers. The bold values signify values <0.05.

**Table 2:** Determinants of mortality in patients with acute kidney injury.

Characteristic	Categories	Mortality *n* (%)		*P* value	Adjusted *P* value	Adjusted OR (95% CI)
		Yes	No			
Age OR (95% CI)		0.93 (0.91–0.96)		<0.001	**<0.001**	0.943 (0.914–0.973)

Comorbidities	Diabetes pt.	22 (16.4)	112 (83.6)	0.225	Not applied	Not applied
	Nondiabetic pt.	14 (11.2)	111 (88.8)			
	CKD	12 (17.4)	57 (82.6)	0.32	Not applied	Not applied
	Non-CKD	24 (12.6)	166 (87.4)			
	Heart disease	18 (23.4)	63 (57.6)	0.014	**0.003**	0.305 (0.137–0.677)
	No heart disease	18 (10.1)	160 (89.9)			

Complication	Hyperkalemia	27 (18.1)	122 (81.9)	0.02	0.123	1.962 (0.833–4.621)
	No hyperkalemia	9 (8.2)	101 (91.8)			
	Hyperphosphatemia	14 (19.4)	58 (80.6)	0.1	Not applied	Not applied
	No hyperphosphatemia	22 (11.8)	165 (88.2)			
	Hypermagnesemia	13 (19.4)	54 (80.6)	0.13	Not applied	Not applied
	No hypermagnesemia	23 (12)	169 (88)			
	Metabolic acidosis	6 (33.3)	12 (66.7)	0.01	0.073	2.993 (0.901–9.938)
	No metabolic acidosis	30 (12.4)	211 (87.6)			

Stage	Stage 1	8 (12.7)	55 (87.3)	0.4	Not applied	Not applied
	Stage 2	8 (10.5)	68 (89.5)			
	Stage 3	20 (16.7)	100 (83.3)			

The bold values signify values <0.05.

**Table 3:** Determinants of the association between patient characteristics and need for renal replacement therapy.

Characteristic	Categories	Renal replacement therapy *n* (%)		*P* value	Adjusted *P* value	Adjusted OR (95% CI)
		Yes	No			
Complication	Hyperkalemia	34 (22.8)	115 (77.2)	0.01	0.435	0.610 (0.176–2.112)
	No hyperkalemia	12 (10.9)	98 (89.1)			
	Hyperphosphatemia	20 (27.8)	52 (72.2)	0.009	**0.045**	0.324 (0.107–0.976)
	No hyperphosphatemia	26 (13.9)	161 (86.1)			
	Hypermagnesemia	13 (19.4)	54 (80.6)	0.6	Not applied	Not applied
	No hypermagnesemia	33 (17.2)	159 (82.8)			
	Metabolic acidosis	12 (66.7)	6 (33.3)	<0.001	**0.016**	0.180 (0.044–0.726)
	No metabolic acidosis	34 (14.1)	207 (85.9)			

Stage	Stage 1	6 (9.5)	57 (90.5)	<0.001	Reference	
	Stage 2	3 (3.9)	73 (96.1)		0.149	12.85 (0.401–411.41)
	Stage 3	37 (30.8)	83 (69.2)		**0.019**	38.773 (1.836–818.59)

Drug during admission	NSAIDS	17 (14.9)	97 (85.1)	0.2	Not applied	Not applied
	No NSAIDs	29 (20)	116 (80)			
	Antibiotic	27 (17.2)	130 (82.8)	0.7	Not applied	Not applied
	No antibiotic	19 (18.6)	83 (81.4)			
	ACE-I/ARBs	11 (20)	44 (80.0)	0.6	Not applied	Not applied
	No ACE-I/ARBs	35 (17.2)	169 (82.8)			
	Diuretic	30 (23.6)	98 (76.4)	0.01	0.956	1.031 (0.344–3.095)
	No diuretic	16 (12.1)	116 (87.9)			

Serum creatinine (mg/dl) (IQR)		0.5 (0.44–0.66)		<0.001	**<0.001**	2.216 (1.398–3.514)

NSAIDs: nonsteroidal anti-inflammatory drugs, angiotensin-converting enzyme inhibitor/angiotensin receptor blockers, antibiotics (cephalosporin, aminoglycoside, bacitracin, and vancomycin), diuretics (furosemide, serum creatinine = the highest level registered during the same admission). The bold values signify values <0.05.

## Data Availability

The data are available within the script, and additional data will be provided if needed.
